# Faster HIV-1 Disease Progression among Brazilian Individuals Recently Infected with CXCR4-Utilizing Strains

**DOI:** 10.1371/journal.pone.0030292

**Published:** 2012-01-26

**Authors:** Maria Cecilia Araripe Sucupira, Sabri Sanabani, Rodrigo M. Cortes, Maria Teresa M. Giret, Helena Tomiyama, Mariana M. Sauer, Ester Cerdeira Sabino, Luiz Mario Janini, Esper Georges Kallas, Ricardo Sobhie Diaz

**Affiliations:** 1 Infectious Diseases Division, Federal University of Sao Paulo, Sao Paulo, Brazil; 2 Sao Paulo Blood Bank, Fundacao Pro-Sangue, Sao Paulo, Brazil; 3 Division of Clinical Immunology and Allergy, University of Sao Paulo, Sao Paulo, Brazil; 4 Infectious Diseases Division, University of Sao Paulo, Sao Paulo, Brazil; 5 Microbiology Division, Federal University of Sao Paulo, Sao Paulo, Brazil; German Primate Center, Germany

## Abstract

**Introduction:**

Primary HIV infection is usually caused by R5 viruses, and there is an association between the emergence of CCXR4-utilizing strains and faster disease progression. We characterized HIV-1 from a cohort of recently infected individuals in Brazil, predicted the virus's co-receptor use based on the *env* genotype and attempted to correlate virus profiles with disease progression.

**Methods:**

A total of 72 recently infected HIV patients were recruited based on the Serologic Testing Algorithm for Recent HIV Seroconversion and were followed every three to four months for up to 78 weeks. The HIV-1 V3 region was characterized by sequencing nine to twelve weeks after enrollment. Disease progression was characterized by CD4+ T-cell count decline to levels consistently below 350 cells/µL.

**Results:**

Twelve out of 72 individuals (17%) were predicted to harbor CXCR4-utilizing strains; a baseline CD4<350 was more frequent among these individuals (p = 0.03). Fifty-seven individuals that were predicted to have CCR5-utilizing viruses and 10 individuals having CXCR4-utilizing strains presented with baseline CD4>350; after 78 weeks, 33 individuals with CCR5 strains and one individual with CXCR4 strains had CD4>350 (p = 0.001). There was no association between CD4 decline and demographic characteristics or HIV-1 subtype.

**Conclusions:**

Our findings confirm the presence of strains with higher *in vitro* pathogenicity during early HIV infection, suggesting that even among recently infected individuals, rapid progression may be a consequence of the early emergence of CXCR4-utilizing strains. Characterizing the HIV-1 V3 region by sequencing may be useful in predicting disease progression and guiding treatment initiation decisions.

## Introduction

HIV-1 disease progression, as reflected by either CD4+ T cell decline or opportunistic diseases, may be related to host and/or virus characteristics. The observation of the natural history of HIV-1 infection in well-characterized cohorts established before the antiretroviral treatment era indicates that the mean time of progression to AIDS is 10 years, although AIDS can develop in as little as two years in a proportion of patients [1]. On the other hand, a proportion of HIV-infected individuals, so-called elite controllers, will not show any CD4 decline over time due to extremely low levels of virus replication, which does not necessarily prevent the HIV-related cell activation or an accelerated aging process [2].

Certain host features are recognized as the main driving force behind disease progression or virus evolution. For example, the CCR5 allele polymorphism in individuals presenting heterozygous deletion of 32 nucleotides (delta 32) is associated with slower disease progression [3] and even better immunologic response to antiretroviral treatment [4]. The same low rates of CD4+ T cell decline are observed in individuals presenting the CCR2-64I mutation [5] or other specific class-I and -II HLA alleles that may have a negative or positive impact on HIV-1 disease progression [6]. Other host-related factors associated with HIV-1 disease progression include a polymorphism at the SDF1-3′A conserved segment of the 3′ untranslated region of the SDF-1 structural gene transcript, which, in homozygous individuals (SDF1-3′A/3′A), is associated with delayed onset of AIDS [7]. Less clear however, is the relationship between co-infections with some other pathogens that may either increase or decrease cell activation, although co-infection with GBV, which is associated with decreased cell activation [8], is more clearly associated with slower disease progression [9] and better rates of antiretroviral response [10].

The influence of HIV-1 genetic diversity on viral evolution and disease progression has also been recognized. Over time, there is an association between the emergence of CXCR4 tropic viruses and faster disease progression [11]. However, although primary infection is caused by viruses that exclusively use the CCR5 co-receptor, infection by dual-tropic viruses may be associated with rapid disease progression [12]. It has been reported that HIV evolves in a host-specific manner, and even among individuals infected with the same viral strain, disease progression may differ, with the emergence of CXCR4-tropic viruses being neither homogeneous nor predictable [13]. Although controversial, biological differences have also been demonstrated between HIV types/subtypes. It has been documented that HIV replication, transmission, cell activation, and disease progression are lower in HIV-2 infection compared to HIV-1 infection [14]. Interestingly, duplication in the NF-κB site, which has been associated with increased pathogenesis in HIV-1 subtype C [15], was also associated with rapid disease progression in one patient infected with HIV-2 [16]. Furthermore, it has also been reported that disease progression among individuals infected with subtypes D and C is faster than in those infected with subtypes A and A/G in Africa [17], and that subtype D infection leads to faster rates of CD4 cell decline and subsequent virological failure compared to infection with clade B and other non-clade B HIV strains in England [18]. It may be conceivable as well that the genetic diversity of the viruses may influence the pace of HIV-1 tropism change because the emergence of X4 viruses occurs very early among subtype D-infected individuals, and it occurs late in infections caused by subtype C viruses [19], [20],[21].

HIV epidemics in Brazil present co-circulating HIV-1 subtypes B, F and C, and few Circulating Recombinant Forms. According to predictions using Bayesian Markov chain Monte Carlo methods and the Reversible-jump MCMC method, HIV-1 subtype B emerged in 1971, subtype F emerged in 1981, BF recombinants emerged in 1989, subtype C emerged in 1987, and BC recombinants emerged in 1992 [22]. There is also a sub-lineage of subtype B harboring the GWGR motif instead of the GPGR motif at the tip of the V3 loop, which was already detectable early in the course of the Brazilian epidemics, in 1983 [23]. Additionally, some reports have suggested that this Brazilian strain (subtype B″) will lead to a slower pace of disease progression than the usual North American/European subtype B present in Brazil [24], [25].

In this study, we characterized HIV-1 from a cohort of recently infected individuals in Brazil and attempted to correlate the rate of CD4+ T cell decline with the genetic diversity of infecting HIV strains, including predicted co-receptor use and/or infection by subtype B″. It has been demonstrated that the specificity of genotype-based predictions of HIV-1 CXCR4 use is usually high [26], [27], as is the specificity of predictions of virologic response to certain CCR5 inhibitors [28].

## Results

Seventy-two individuals were initially included in the cohort. Seventy (94.6%) were male, 90% were men who have sex with men and none had acquired HIV through blood exposure. The mean age was 32.7 years, ranging from 20 to 56, the mean viral load was 72,552 (80,443) copies/mL (4.9 log_10_), ranging from <400 to 703,000 copies/mL (<2.6 to 5.8 log_10_), and the mean CD4+ T cell count was 573 cells/µL, ranging from 83 to 2,449 cells/µL. It is described here the analysis for the prediction of co-receptor use using false positive rate of 10%, since it correlated best with disease progression in this group of patients. According to the V3 region profile, 12 out of 72 individuals (17%) were predicted to harbor CXCR4-utilizing strains, as predicted by the bioinformatics tool Geno2pheno[coreceptor]. According to the lab tests results from the first visit, 5 out of the 12 patients who were predicted to be infected with CXCR4-utilizing HIV strains and 8 out of the 60 who were predicted to be infected with CCR5-utilizing strains presented with baseline CD4+ T cell counts lower than 350 cells/µL (p = 0.03, Fisher's exact test). Table 1 depicts the demographic and virologic/immunologic characteristics of individuals infected with R5 or CXCR4-tropic viruses.

**Table 1:** Characteristics of individuals infected by different tropic HIV.

		R5	CXCR4 using strains	*p*
**Caracteristics**		60 (83.3%)	12 (16.7%)	
Gender	Male	58 (96.7%)	10 (83.3%)	0.13
Exposure	MSM*	53 (88.3%)	9 (75.0%)	0.35
Age (mean)		32.65 (±1.06)	33.08 (±3.30)	0.88
Basal Viral Load (log mean)		4.277 (±0.11)	4.404 (±0.28)	0.65
Basal CD4 count (mean)		566.0 (±27.50)	611.7 (±184.70)	0.65
Tip of V3 loop	B	40	6	0.44
	B″	20	6	
Subtype	B	56	12	0.47
	non-B	4	0	
CCR5 Δ32		4 (6,7%)	1 (8,3%)	
GBV-C		17 (28,3%)	2 (16,7%)	
HLA Allele B	27	5 (8,3%)	0	
	57	4 (6,7%)	2 (16,7%)	
	58	4 (6,7%)	2 (16,7%)	
NRTI and PI Primary Resistance		5	1	0.74

*Men who have sex with men.

At the tip of V3 loop, strains were classified as B if they harbor a proline or B″ if they harbor a tryptophan or a related amino acid.

Five R5-infected individuals presented transmitted drug-resistant strains, compared to one CXCR4-tropic HIV-infected individual, for a prevalence of 8.3%. Mutations were as follows: K103N (1 X4), D30N (1), L10I/A71T+M184V+K103N (1), K103N/Y181C (1), M184I (1), K103n+M184V (1). As seen in Table 1, there were no differences in the mean HIV-1 baseline viral load, mean baseline CD4 count, age, HIV-1 subtype, gender, route of HIV-1 transmission, and transmitted drug-resistant strains between individuals infected by R5- or CXCR4-utilizing viruses.

**Figure 1:**
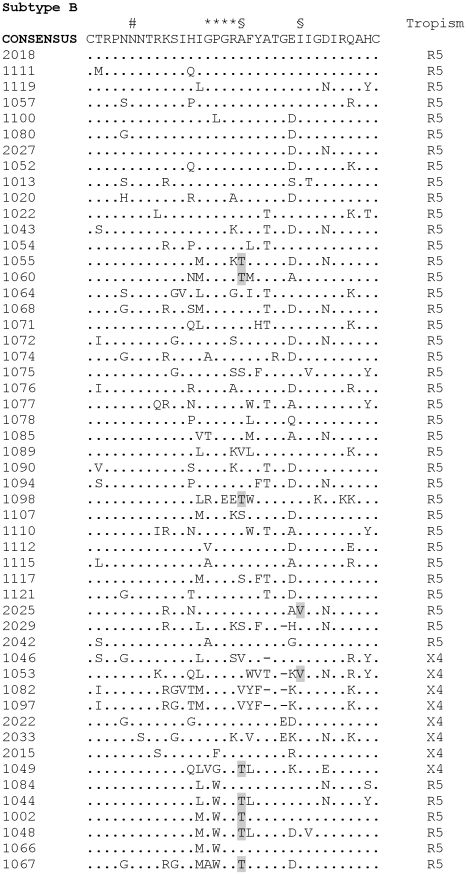
Amino acid alignment of V3 sequences. Subtype B sequences were aligned with the consensus sequence. Amino acid positions appear at the top of each alignment, with dots indicating identities whereas dashes indicate deletions. # indicates the N-linked glycosylation site, * indicates the GPGR motif at the tip of the V3 loop, and § indicates positions 316 and 323 (HXB2 positions), which are associated with in vitro selection of maraviroc resistance (316T and 323 V substitutions are shaded) [29]. Inferred tropism is indicated to the right of each sequence (CXCR4-using strains are named X4 for the sake of simplicity).

As seen in Figure 1, 68 individuals were infected with subtype B, according to the V3 sequence, two were infected with subtype F, one with subtype C, and one with subtype A, which clustered closer to CRF_2 sequences (data not shown). Forty-two clade B-infected individuals harbored a proline at the tip of the V3 loop (GPG motif), whereas 18 harbored a tryptophan (GWG, the so called B″ lineage), and eight other samples presented different amino acids, including phenylalanine (2), leucine, glycine, methionine, threonine and valine (1 each). All these alternative amino acids are encoded by nucleotide triplets that are closer to the one encoding tryptophan (TGG) than the ones that encode proline at this position in this specific group of patients (CCA/G/T), thus suggesting that they are derived from the B″ lineage. The A316T substitution was detected in 15 individuals, whereas the I323V substitution was detected in only two subjects; both mutations were selected for by maraviroc *in vitro*, which lead to a plateau in the PhenoSense HIV Entry Assay (Monogram Biosciences, CA), preventing maximal inhibition by maraviroc [29] (Figure 1). Interestingly, 12 out of the 15 cases presenting A316T substitution appeared among the 26 B″-related strains, compared to three out of the 42 clade B strains (Fisher's exact test p = 0.0003).

**Figure 2:**
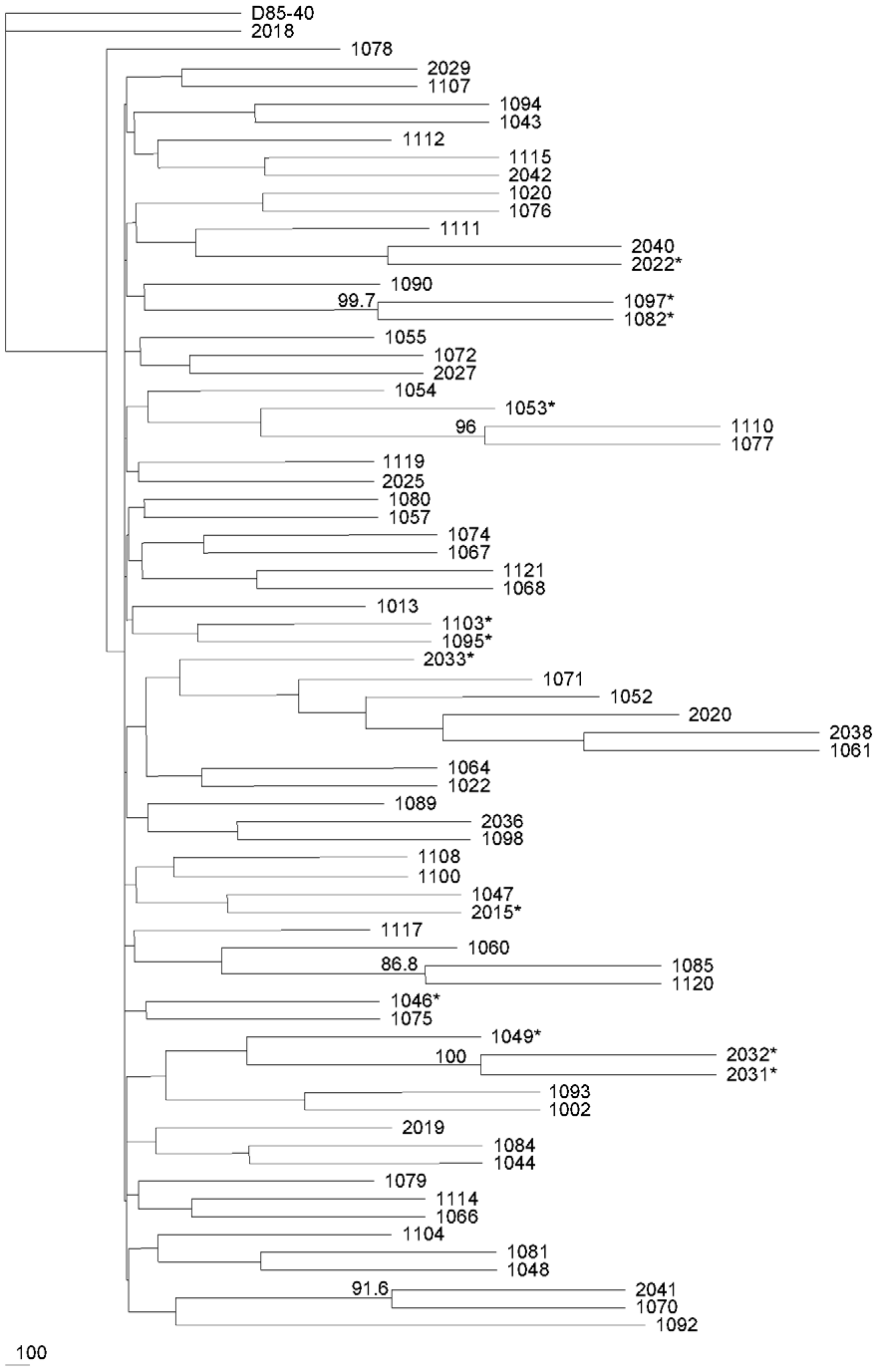
Phylogenetic relationships between analyzed samples. D85_40 at the top is the North-American/European B consensus sequence. * indicates CXCR4-using strains. Bootstrap values above 85% are indicated at the intersections of branches.

Phylogenetic relationships using all of the samples revealed only one pair of patients presenting evidence of clustering based on the low bootstrap values (below 98%, as previously suggested) [30]. This indicates that the majority of individuals analyzed were epidemiologically unrelated, and clear evidence of convergence towards CXCR4 sequences was not detected (Figure 2).

Overall, the mean and median CD4+ T cell counts were 551.5 and 529 cells/µL, respectively at baseline, and 537.9 and 475 cells/µL, respectively at visit 6. These CD4+ T-cell counts represented a mean and median declines of −13.6 and −54 cells from baseline over this 78-week period (visit number 6). Specifically, the mean CD4 decline was −5.6 cells/µL for R5-infected individuals, compared to −168.9 cells/µL among individuals infected with CXCR4-utilizing strains. The Cox proportional-hazards regression revealed a risk ratio for CD4+ T cell count decline to a level below 350 cells/µL of 0.99 for higher baseline CD4 T cell count (protective effect, p = 0.0001) and 5.1 for X4 strains (p = 0.007). When age was kept in the model, the age risk ratio was 1.1 with a borderline p value of 0.058, but the risk ratio for X4 viruses increased to 10.1 (p = 0.001) whereas the risk ratio for high baseline CD4 remained at 0.99 (p = 0.007), thus suggesting that age enhances the effect of X4 strains on CD4 decline over time.

The mean CD4+ T cell count at baseline among individuals with baseline viral loads above 100,000 copies/mL (after visit 2) was 434.7 cells/µL, compared to 572.5 cells/µL in individuals with baseline viral loads below 100,000 copies/mL (Mann-Whitney p = 0.046). At visit number 6, mean CD4+ T cell counts were 489 versus 543.5 for patients with viral loads >100,000 and <100,000, respectively (Mann-Whitney p = 0.56). Interestingly, there was no statistically significant difference in CD4 T cell decline between these two groups over this short period of time; the CD4 T cell count evolution slope was +0.34±0.55 for patients with >100,000 copies/mL, compared to −0.43±1.3 among individuals with baseline viral loads <100,000 copies/mL. We hypothesize that the initial impact on the CD4 T cell count among individuals with higher viral loads followed by partial recovery of CD4+ T cells after reaching the set point may obscure the impact of persistent higher viral loads (i.e.; >100,000 copies/mL) in the CD4 T cell decay during short term follow-up.

**Figure 3:**
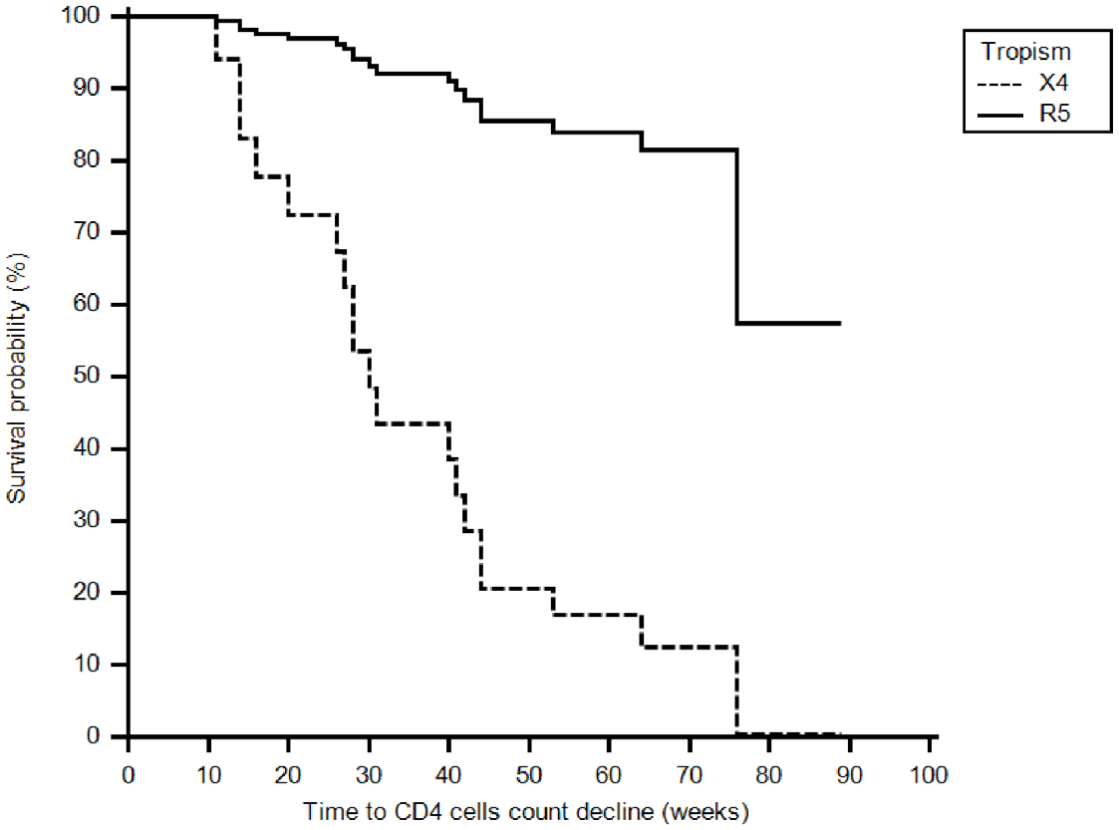
Associations between inferred tropism and time to CD4 decline to levels below 350 cells/µL (survival probability). There was a statistically significant association between the presence of CXCR4-using viruses (X4) in the genotype at baseline and an earlier time to CD4 decline below 350 cells/µL (log rank P = 0.0450). The mean time to CD4 decline below 350 was 51.31±3.66 and 34.14±10.61 months for R5- and X4-infected individuals, respectively.

**Figure 4:**
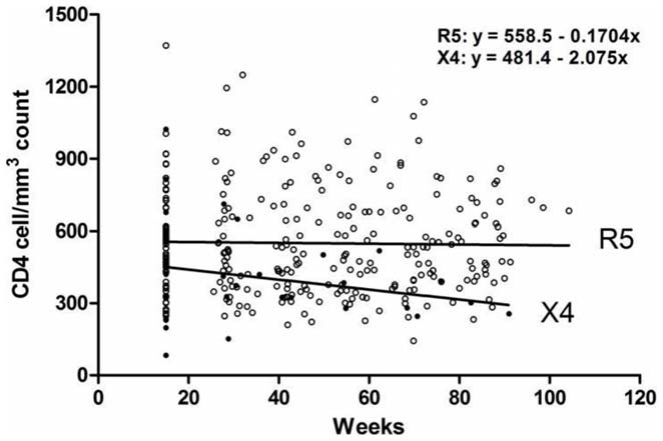
CD4 decline over time in individuals infected with R5 or CXCR4-using viruses (mean initial CD4+ T cell counts of 558.5 and 481.4 respectively).

Fifty-seven individuals who were followed for up to 78 weeks and presented CD4+ T-cell counts above 350 cells/µL at baseline were evaluated and included in the disease progression analysis (Figure 3). Forty-seven individuals predicted to have CCR5 strains and 10 individuals predicted to have CXCR4 strains presented with baseline CD4>350 cells/µL; after 78 weeks, 33 patients with R5 strains and 1 patient with CXCR4 strains had a CD4 count >350 cells/µL (p = 0.001, Fisher's exact test). As seen in Figure 4, the slope of CD4 decline was minus 2.07 for individuals with CXCR4-utilizing viruses, compared to minus 0.17 for R5-infected individuals. The probability of CD4 decline from above to below 350 cells/µL over time was similar for individuals infected with clade B and clade B″ viruses (Fisher's exact test p = 0.5). There was no association between CD4+ T-cell count decline to below 350 cells/µL over time and demographic characteristics, HIV-1 subtype, gender, or baseline viral loads.

Overall, five individuals presented the CCR5 Δ32 allele (6.9%), 19 presented GBVC viremea (26.4%), 5 presented HLA_B*27 (6.9%), 6 presented HLA_B*57 and 6 HLA_B*58, and none of these variable were statistically correlated to the CD4 decline or viral tropism.

## Discussion

We have established a well-characterized cohort of recently infected individuals, and therefore, we were able for the first time to describe the natural progression of HIV disease in Brazil based on CD4+ T cell decline over time *vis a vis* the genetic characterization of the infecting viruses. Individuals coming from five counseling and testing sites in the city of São Paulo were recruited from 2002 to 2007 after being detected as having recent HIV infection according to the STAHRS definition. To maximize the odds of true recent HIV infection based on the laboratory results, we confirmed that recruited individuals were able to fully seroconvert in the follow-up visits, thus becoming seropositive for the less sensitive STAHRS assay. Using this strategy, we analyzed 72 individuals in which the HIV-1 V3 region of gp120 was characterized from samples obtained during the second visit, approximately nine to 12 weeks after recruitment. We also have been able to demonstrate that CXCR4-utilizing viruses were predicted to be present in 12 out of 72 individuals (17%) at that time, which is in accordance with the range described in the literature (4% to 15.9%) [31], [32], [33].

It has been consistently reported that individuals are initially infected with CCR5-utilizing strains. It has been recognized using mathematical models that 75% of individuals will be infected by only one HIV-1 strain whereas the remaining 25% will be infected by two to five HIV strains [34]. This genetic restriction occurs in spite of the transmission route, and it is so significant that only one variant may be selected from several quasispecies from different blood donors after the transfusion of platelets coming from different HIV-infected individuals [35]. Therefore, we believe that the V3 sequence profiles characterized in our study are an accurate representation of the homogeneous quasispecies present at that stage of HIV infection. It is also known that the change in HIV-1 tropism from R5 to X4 receptors generally occurs late in the disease when viruses start to preferentially replicate in the thymus rather than in the gastrointestinal tract, and the detection of X4 strains is correlated with rapid disease progression. (reviewed in reference [33]) Detection of CXCR4-tropic strains early in the course of disease may be attributed to faster evolution from initial R5 to X4 viruses or transmission of dual-tropic viruses, and we believe that these two possibilities may justify the high proportion of CXCR4-tropic viruses found in our cohort.

Not surprisingly, we were able to observe the association between the presence of CXCR4-utilizing strains and faster disease progression as characterized by CD4+ T cell counts below 350 cells/µL. Although baseline CD4+ T cell count was not statistically different between the two groups, the decrease to levels below 359 cells/µL could somewhat be biased by the slightly lower baseline levels in subjects with predicted CXCR4 using viruses (566 versus 611 cells/µL). It is unclear whether the association of faster disease progression with CXCR4-using strains represent a cause or a consequence of HIV-1-related immune suppression. However, the detection of CXCR4-using strains during recent infection in a group of patients with relatively higher CD4+ T cell levels and the observation of the steepest CD4+ T cell decline among these individuals may indicate that this phenomenon is more likely to be the cause rather than a consequence of immune suppression. Therefore, our findings stress the possibility of the presence of strains with more alleged *in vitro* pathogenicity during the early phases of HIV infection, and also suggest that, even among recently infected individuals, rapid progression may be a consequence of the early emergence of either X4 or dual-tropic HIV strains.

We have not been able to correlate the pace of disease progression and the presence of the Brazil-specific HIV-1 B strains harboring a tryptophan residue at position 16 at the tip of the V3 loop (the so-called B″ strains), as had been previously suggested [24], [25]. It is interesting to note that B″ strains emerged very early in the Brazilian epidemics, accounting for approximately 50% of subtype B strains in Brazil [ref] [23]. It is also interesting that the prevalence of B″ strains is lower among patients in lower CD4+ T cell count strata, whereas the prevalence of the classical GPGR strains remains unchanged and the prevalence of strains presenting other amino acids at the tryptophan position inceases (V3 position 16). Sera sample from patients harboring most of these strains harboring other amino acids at position 16 will react with specific B″ peptides and not with GPGR peptides, thus suggesting that they have evolved from B″ strains [23]. As tryptophan is coded by the TGG nucleotide triplet, it is conceivable that the common hypermutation process observed in HIV (G to A substitution) may lead to a disappearance of tryptophan over time because TGA, TAG and TAA are stop codons; therefore, tryptophan may be replaced by other related amino acids. One possible explanation for the sustained prevalence of B″ strains in the Brazilian population may be related to the fact that most HIV transmission occurs during the first years of infection, a time when B″ strains are still present in an infected individual. We therefore think that the lack of correlation between slower disease progression and B″ strains found in our study is accurate because by the time the disease progresses in B″-infected individuals, the tryptophan residue may have already been replaced by other amino acids. One interesting finding was the high prevalence of A316T substitution at the V3 region (HXB2 position) in this set of samples. As previously mentioned, these mutations have been selected by maraviroc in cell culture, leading to decreased susceptibility to this drug [29]. The impact of these mutations has been confirmed by reverse mutation, which restored wildtype susceptibility to maraviroc. However, this mutation has been reported to be exceedingly rare in clade B viruses, accounting for only two out of 23,000 sequences in the Los Alamos HIV sequence database [29]. Another interesting finding was the association of A316T substitution with the Brazilian clade B strains (B″), which are extremely common in Brazil and rare elsewhere. Therefore, the phenotypic impact of this substitution on maraviroc susceptibility in B″ strains deserves further confirmation.

We also investigated the influence of other co-factors related to HIV-1 disease progression such as the CCR5 polymorphism, GBVC viremea, and presence of “protective” HLA profiles such as HLA B*27, B*57 and/or B*58 and were not able to find any statistic correlation with CD4+ T cell decline, perhaps due to the small number of patients here analyzed.

We recognize that this study may lack sensitivity and/or specificity in detecting patients infected by X4 or dual-tropic/mixed HIV populations because phenotypic assays were not used in this study. However, we achieve the objective of predicting genotypic correlates of HIV-1 disease progression using available tools. Our findings confirm the similar results of another recent study [36], and we believe that our findings demonstrate the necessity of trying to predict HIV-1 co-receptor use among recently infected/diagnosed naïve individuals because it may predict which individuals will be more likely to progress faster in their HIV-related immune deficiency, and it may perhaps suggest that a safer approach in dealing with these individuals could be an earlier initiation of antiretroviral therapy. For the purpose of this sort of HIV-1 characterization, genotype-based techniques may prove to be more cost-effective than phenotypic assays because they are less cumbersome, easily available, and less expensive.

## Methods

### Ethics Statement

Informed written consent was obtained from all the patients and the study was approved by the Ethics Committees and the Institutional Review Board of the Federal University of Sao Paulo [#0919/01].

### Cohort characterization

A cohort of individuals recently infected with HIV was initiated in May 2002. Individuals seeking free and anonymous testing and counseling services offered by the City of São Paulo Health Department and identified as infected with HIV-1 were offered to undergo the Serologic Testing Algorithm for Recent HIV Seroconversion (STARHS) after signing an initial IRB-approved informed consent. They were invited to join the cohort if recent HIV infection was detected (see below). As of May 2002, we were able to recruit 72 individuals in which *env* V3 characterization was possible. Recruited individuals were followed every three to four months after the initial clinical visit [37].

### Characterization of recent HIV-1 infection using the Serologic Testing Algorithm for Recent HIV Seroconversion (STARHS)

The Vironostika® HIV-1 Micro-ELISA System (bioMérieux Inc., Durham, NC, USA) was used to retest the original HIV-positive samples. The Vironostika LS/EIA tests a 1∶20,000 dilution of the specimen under modified incubation conditions. Specimens found to be positive on the S/EIA and negative on the LS/EIA are considered to represent recent infection [38], [39]. This method is based upon the slow rate of increase in antibody titers observed during the early period of infection, as well as on data from study subjects with known dates of seroconversion. Based on the calibration of the assays and on a defined threshold, the serologic testing algorithm for recent HIV seroconversion (STARHS) classifies HIV infection as recent or long-standing depending on the differential HIV antibody titer. The assay performs uniformly on HIV-1 subtype B, and the mean window period between seroconversion on the S/EIA and seroconversion on the LS/EIA is 170 days, with a 95% confidence interval (95% CI) of 145–200; we therefore estimated incidence based on the 170-day period. The STARHS was repeated in samples collected six months after the initial visit to document full seroconversion and to confirm the true positive nature of recent HIV infections the enrolled individuals, since 2% of the cases can be falsely classified to be seroconverters. Additionally, false recency rate (FRR) is population specific occurring among AIDS cases, elite controllers, and HAART suppressed patients in the population under study, which were not the case the studied population [40], [41]. All testing was performed at the Retrovirology Laboratory of the Federal University of São Paulo, located in São Paulo, Brazil. Volunteers were also seen by an Infectious Diseases physician to determine whether the clinical and laboratory evaluation was compatible with a recent infection.

### HIV extraction, amplification, and sequencing

We selected proviral DNA rather than RNA given that, for HIV-1 tropism evaluation, proviral DNA may constitute a more sensitive method to detect CXCR4-utilizing strains [26]. Furthermore, as the patients in this study were evaluated at a time point close to primary infection, the divergence between these two compartments would not have been significant. Samples analyzed were from the second visit, which occurred approximately 9 to 12 weeks after enrollment in the cohort. Proviral HIV-1 DNA was purified using the QIAamp blood kit (Qiagen, Santa Clarita, CA, USA), in accordance with the manufacturer's instructions. To analyze the V3 region of gp120 I, a 1.2-kb fragment of V1–V5 was amplified in the first round and a 350-bp fragment of C2V3C3 was amplified in the second PCR, as previously described [42]. The protease and reverse transcriptase regions of the *pol* gene were also amplified to assess primary HIV-1 resistance to antiretrovirals, as previously described [43]. Purified PCR products were sequenced bi-directionally with an ABI PRISM BigDye Terminator Cycle Sequencing Ready Reaction Kit and Ampli Taq DNA Polymerase (Applied Biosystems, Foster City, CA, USA). Sequences were corrected and assembled using the Sequencher 4.0 program (Genecodes, Ann Arbor, MI, USA).

### Sequencing analysis and prediction of co-receptor use

The computer-modeled amino acid sequences were aligned and used as a guide for manual editing of nucleotide sequence alignments. The alignments were generated using the CLUSTAL X program, version 3.0 [44]. For each alignment, phylogenetic analyses were performed using the PHYLIP program package, version 3.57. The DNAdist program was used to calculate distance matrices based on the maximum-likelihood model, and neighbor-joining trees were generated using the Neighbor and Consense programs. Statistical significance was assessed with bootstrap tests in a total of 100 replications. (GenBank accession numbers pending). Co-receptor use was predicted using the bioinformatics tool Geno2pheno[coreceptor] (false positive rate of 5, 10, and 20% - Table S1), coreceptor.bioinf.mpi-inf.mpg.de [27]. We chose to pursue the analysis using false positive rate of 10% which best correlated with disease progression in this group of patients.

### Antiretroviral resistance analysis

Mutations related to resistance to NRTI, NNRTI, and PI antiretrovirals were evaluated according to published guidelines, which excluded common polymorphisms [45]. The prevalence of mutations A316T and I323V in the V3 loop of gp120 (HXB2 positions), which display selection by treatment with maraviroc *in vitro* and are related to decreased susceptibility to this inhibitor in R5 strains, was also evaluated [29].

### Detection and quantification of GB virus type C RNA

Viral RNA was extracted and reverse transcribed and a fragment of the nonstructural 5a region (NS5a) was PCR amplified as previously [46], [47], [48] described. After amplification, 5 ml of the PCR product was used for electrophoresis analysis on a 2% agarose gel. The positive and negative samples were corroborated with nested RT_PCR that amplified a fragment of 344 bp of the 50 noncoding region (50 NCR) as previously described. After amplification, 5 ml of the PCR product was used for electrophoresis analysis on a 2% agarose gel.

### CCR5 polymorphism

We obtained genomic DNA samples extracted from 300 µl of buffy coat using a QIAamp Blood Kit (QIAGEN Inc, CA), using the methodology indicated by the manufacturer. The presence of CCR5Δ32, representing the heterozygous status of the allele by the host was determined by polymerase chain reaction (PCR) as previously described [49]. Subsequently, amplified products were separated with electrophoresis in a 3% agarose gel for 40 minutes at 110 mV and visualized with ethidium bromide under ultraviolet light. The expected PCR product size was 241 bp for the wild- type and 209 bp for the CCR5Δ32 alleles.

### HLA class I genotyping

For each subject a blood sample was taken and genomic DNA was extracted using a QIAamp DNA blood minikit (Qiagen). All samples were analyzed for *HLA-A*, *B and C* alleles by reverse polymerase chain reaction (PCR) sequence-specific oligonucleotide (SSO) reverse hybridization on microbead arrays (Luminex ™ technology; Luminex Corporation, Austin, TX) after locus-specific amplification on genomic DNA samples, using LABType® SSO reagents (One Lambda, INC. Canoga Park, CA, USA). This assay provided results at an intermediate level (groups of alleles), with assignment of the four-digit allele in some cases.

### Characterization of disease progression and statistical analysis

All included individuals were followed at the HIV outpatient clinics of the Federal University of São Paulo. Clinical data, RNA-HIV-1 viral loads and CD4+ T cell counts were collected every three to four months. Disease progression was characterized by the decrease of CD4+ T cell counts from above 350 cells/µL to levels consistently below 350 cells/µL, and/or the initiation of antiretroviral treatment. Antiretroviral treatment was initiated according to the Brazilian Guidelines for HIV-1 Therapy (www.aids.gov.br), which until 2009, recommended treatment for patients with CD4+ T cell counts below 350 cells/µL or in the presence of AIDS-defining conditions. The level of CD4 bellow 350 cells/µL was arbitrarily chosen since all individuals that reached these levels started with antiretroviral treatment, thus obscuring the disease progression end-point. On the other hand, in this specific group of patients, antiretroviral treatment was not initiated in any individual with CD4+ T-cell levels above 350 cells/µL. Statistical analyses were performed using the Chi-square and Fisher's exact test. The influence of the HIV-1 V3 sequence on clinical outcomes was assessed by Kaplan–Meier analyses. Cox proportional hazard regression was used to calculate univariate and multivariate risk ratios (RR) and 95% confidence intervals (CI). Baseline variables examined included HIV-1 baseline viral load, CD4+ T cell count, age, HIV-1 subtype, gender, and route of HIV-1 transmission.

## Supporting Information

Table S1 Tropism Prediction using genotopheno [coreceptor] with false positive rates (FPR) of 5, 10 and 20%.
(DOC)

## References

[1] Mellors JW, Margolick JB, Phair JP, Rinaldo CR, Detels R (2007). Prognostic value of HIV-1 RNA, CD4 cell count, and CD4 Cell count slope for progression to AIDS and death in untreated HIV-1 infection.. JAMA.

[2] Hunt PW, Brenchley J, Sinclair E, McCune JM, Roland M (2008). Relationship between T cell activation and CD4+ T cell count in HIV-seropositive individuals with undetectable plasma HIV RNA levels in the absence of therapy.. J Infect Dis.

[3] Dean M, Carrington M, Winkler C, Huttley GA, Smith MW (1996). Genetic restriction of HIV-1 infection and progression to AIDS by a deletion allele of the CKR5 structural gene. Hemophilia Growth and Development Study, Multicenter AIDS Cohort Study, Multicenter Hemophilia Cohort Study, San Francisco City Cohort, ALIVE Study.. Science.

[4] Accetturi CA, Pardini R, Novaes Pinto GH, Turcato G, Lewi DS (2000). Effects of CCR5 genetic polymorphism and HIV-1 subtype in antiretroviral response in Brazilian HIV-1-infected patients.. J Acquir Immune Defic Syndr.

[5] Ioannidis JP, Rosenberg PS, Goedert JJ, Ashton LJ, Benfield TL (2001). Effects of CCR5-Delta32, CCR2-64I, and SDF-1 3′A alleles on HIV-1 disease progression: An international meta-analysis of individual-patient data.. Ann Intern Med.

[6] Hendel H, Caillat-Zucman S, Lebuanec H, Carrington M, O'Brien S (1999). New class I and II HLA alleles strongly associated with opposite patterns of progression to AIDS.. J Immunol.

[7] Winkler C, Modi W, Smith MW, Nelson GW, Wu X (1998). Genetic restriction of AIDS pathogenesis by an SDF-1 chemokine gene variant. ALIVE Study, Hemophilia Growth and Development Study (HGDS), Multicenter AIDS Cohort Study (MACS), Multicenter Hemophilia Cohort Study (MHCS), San Francisco City Cohort (SFCC).. Science.

[8] Maidana-Giret MT, Silva TM, Sauer MM, Tomiyama H, Levi JE (2009). GB virus type C infection modulates T-cell activation independently of HIV-1 viral load.. AIDS.

[9] Xiang J, Wunschmann S, Diekema DJ, Klinzman D, Patrick KD (2001). Effect of coinfection with GB virus C on survival among patients with HIV infection.. N Engl J Med.

[10] Souza IE, Zhang W, Diaz RS, Chaloner K, Klinzman D (2006). Effect of GB virus C on response to antiretroviral therapy in HIV-infected Brazilians.. HIV Med.

[11] Koot M, Keet IP, Vos AH, de Goede RE, Roos MT (1993). Prognostic value of HIV-1 syncytium-inducing phenotype for rate of CD4+ cell depletion and progression to AIDS.. Ann Intern Med.

[12] Markowitz M, Mohri H, Mehandru S, Shet A, Berry L (2005). Infection with multidrug resistant, dual-tropic HIV-1 and rapid progression to AIDS: a case report.. Lancet.

[13] Diaz RS, Zhang L, Busch MP, Mosley JW, Mayer A (1997). Divergence of HIV-1 quasispecies in an epidemiologic cluster.. AIDS.

[14] Cavaleiro R, Brunn GJ, Albuquerque AS, Victorino RM, Platt JL (2007). Monocyte-mediated T cell suppression by HIV-2 envelope proteins.. Eur J Immunol.

[15] Montano MA, Novitsky VA, Blackard JT, Cho NL, Katzenstein DA (1997). Divergent transcriptional regulation among expanding human immunodeficiency virus type 1 subtypes.. J Virol.

[16] Fusuma EE, Caruso SC, Lopez DF, Costa LJ, Janini LM (2005). Duplication of peri-kappaB and NF-kappab sites of the first human immunodeficiency virus type 2 (HIV-2) transmission in Brazil.. AIDS Res Hum Retroviruses.

[17] Kanki PJ, Hamel DJ, Sankale JL, Hsieh C, Thior I (1999). Human immunodeficiency virus type 1 subtypes differ in disease progression.. J Infect Dis.

[18] Easterbrook PJ, Smith M, Mullen J, O'Shea S, Chrystie I (2010). Impact of HIV-1 viral subtype on disease progression and response to antiretroviral therapy.. J Int AIDS Soc.

[19] Tscherning C, Alaeus A, Fredriksson R, Bjorndal A, Deng H (1998). Differences in chemokine coreceptor usage between genetic subtypes of HIV-1.. Virology.

[20] Ping LH, Nelson JA, Hoffman IF, Schock J, Lamers SL (1999). Characterization of V3 sequence heterogeneity in subtype C human immunodeficiency virus type 1 isolates from Malawi: underrepresentation of X4 variants.. J Virol.

[21] Cecilia D, Kulkarni SS, Tripathy SP, Gangakhedkar RR, Paranjape RS (2000). Absence of coreceptor switch with disease progression in human immunodeficiency virus infections in India.. Virology.

[22] Leal, Eacute, lcio, Martins LO, Janini LM (2008). Evolutionary Dynamics of HIV-1 BF and CB Recombinants and Its Parental Counterparts in South America.. Retrovirology: Research and Treatment.

[23] Diaz RS, Leal E, Sanabani S, Sucupira MCA, Tanuri A (2008). Selective Regimes and Evolutionary Rates of HIV-1 Subtype B V3 Variants in the Brazilian Epidemic..

[24] Casseb J, Komninakis S, Abdalla L, Brigido LF, Rodrigues R (2002). HIV disease progression: is the Brazilian variant subtype B′ (GWGR motif) less pathogenic than US/European subtype B (GPGR)?. Int J Infect Dis.

[25] Santoro-Lopes G, Harrison LH, Tavares MD, Xexeo A, Dos Santos AC (2000). HIV disease progression and V3 serotypes in Brazil: is B different from B-Br?. AIDS Res Hum Retroviruses.

[26] Verhofstede C, Vandekerckhove L, Eygen VV, Demecheleer E, Vandenbroucke I (2009). CXCR4-using HIV type 1 variants are more commonly found in peripheral blood mononuclear cell DNA than in plasma RNA.. J Acquir Immune Defic Syndr.

[27] Sing T, Low AJ, Beerenwinkel N, Sander O, Cheung PK (2007). Predicting HIV coreceptor usage on the basis of genetic and clinical covariates.. Antivir Ther.

[28] McGovern RA, Thielen A, Mo T, Dong W, Woods CK (2010). Population-based V3 genotypic tropism assay: a retrospective analysis using screening samples from the A4001029 and MOTIVATE studies.. AIDS.

[29] Westby M, Smith-Burchnell C, Mori J, Lewis M, Mosley M (2007). Reduced maximal inhibition in phenotypic susceptibility assays indicates that viral strains resistant to the CCR5 antagonist maraviroc utilize inhibitor-bound receptor for entry.. J Virol.

[30] Brenner BG, Roger M, Moisi DD, Oliveira M, Hardy I (2008). Transmission networks of drug resistance acquired in primary/early stage HIV infection.. AIDS.

[31] de Mendoza C, Van Baelen K, Poveda E, Rondelez E, Zahonero N (2008). Performance of a population-based HIV-1 tropism phenotypic assay and correlation with V3 genotypic prediction tools in recent HIV-1 seroconverters.. J Acquir Immune Defic Syndr.

[32] Huang W, Toma J, Stawiski E, Fransen S, Wrin T (2009). Characterization of human immunodeficiency virus type 1 populations containing CXCR4-using variants from recently infected individuals.. AIDS Res Hum Retroviruses.

[33] Frange P, Galimand J, Goujard C, Deveau C, Ghosn J (2009). High frequency of X4/DM-tropic viruses in PBMC samples from patients with primary HIV-1 subtype-B infection in 1996–2007: the French ANRS CO06 PRIMO Cohort Study.. J Antimicrob Chemother.

[34] Keele BF, Giorgi EE, Salazar-Gonzalez JF, Decker JM, Pham KT (2008). Identification and characterization of transmitted and early founder virus envelopes in primary HIV-1 infection.. Proc Natl Acad Sci U S A.

[35] Diaz RS, Sabino EC, Mayer A, deOliveira CF, Mosley JW (1996). Lack of dual HIV infection in a transfusion recipient exposed to two seropositive blood components.. AIDS Res Hum Retroviruses.

[36] Raymond S, Delobel P, Mavigner M, Cazabat M, Encinas S (2010). CXCR4-using viruses in plasma and peripheral blood mononuclear cells during primary HIV-1 infection and impact on disease progression.. AIDS.

[37] Kallas EG, Bassichetto KC, Oliveira SM, Goldenberg I, Bortoloto R (2004). Establishment of the serologic testing algorithm for recent human immunodeficiency virus (HIV) seroconversion (STARHS) strategy in the city of Sao Paulo, Brazil.. Braz J Infect Dis.

[38] Janssen RS, Satten GA, Stramer SL, Rawal BD, O'Brien TR (1998). New testing strategy to detect early HIV-1 infection for use in incidence estimates and for clinical and prevention purposes.. Jama.

[39] Kothe D, Byers RH, Caudill SP, Satten GA, Janssen RS (2003). Performance characteristics of a new less sensitive HIV-1 enzyme immunoassay for use in estimating HIV seroincidence.. J Acquir Immune Defic Syndr.

[40] Busch MP, Pilcher CD, Mastro TD, Kaldor J, Vercauteren G (2010). Beyond detuning: 10 years of progress and new challenges in the development and application of assays for HIV incidence estimation.. AIDS.

[41] Cimerman S, Sucupira MC, Lewi DS, Diaz RS (2007). Less sensitive HIV-1 enzyme immunoassay as an adjuvant method for monitoring patients receiving antiretroviral therapy.. AIDS Patient Care STDS.

[42] Sanabani S, Neto WK, de Sa Filho DJ, Diaz RS, Munerato P (2006). Full-length genome analysis of human immunodeficiency virus type 1 subtype C in Brazil.. AIDS Res Hum Retroviruses.

[43] Sucupira MC, Caseiro MM, Alves K, Tescarollo G, Janini LM (2007). High levels of primary antiretroviral resistance genotypic mutations and B/F recombinants in Santos, Brazil.. AIDS Patient Care STDS.

[44] Thompson JD, Gibson TJ, Plewniak F, Jeanmougin F, Higgins DG (1997). The CLUSTAL_X windows interface: flexible strategies for multiple sequence alignment aided by quality analysis tools.. Nucleic Acids Res.

[45] Bennett DE, Camacho RJ, Otelea D, Kuritzkes DR, Fleury H (2009). Drug resistance mutations for surveillance of transmitted HIV-1 drug-resistance: 2009 update.. PLoS One.

[46] Jarvis LM, Davidson F, Hanley JP, Yap PL, Ludlam CA (1996). Infection with hepatitis G virus among recipients of plasma products.. Lancet.

[47] Schlueter V, Schmolke S, Stark K, Hess G, Ofenloch-Haehnle B (1996). Reverse transcription-PCR detection of hepatitis G virus.. J Clin Microbiol.

[48] Tucker TJ, Smuts H, Eickhaus P, Robson SC, Kirsch RE (1999). Molecular characterization of the 5′ non-coding region of South African GBV-C/HGV isolates: major deletion and evidence for a fourth genotype.. J Med Virol.

[49] Munerato P, Azevedo ML, Sucupira MC, Pardini R, Pinto GH (2003). Frequency of polymorphisms of genes coding for HIV-1 co-receptors CCR5 and CCR2 in a Brazilian population.. Braz J Infect Dis.

