# Supplementary material for: Faster HIV-1 Disease Progression among Brazilian Individuals Recently Infected with CXCR4-Utilizing Strains
Source: PLoS One. 2012 Jan 26;7(1):e30292. doi: 10.1371/journal.pone.0030292 (PMC3266896; doi:10.1371/journal.pone.0030292)
Supplement: Table S1 — Tropism Prediction using genotopheno [coreceptor] with false positive rates (FPR) of 5, 10 and 20%. (DOC) [file pone.0030292.s001.doc]

**Table S1:** Tropism Prediction using genotopheno [coreceptor] with false positive rates (FPR) of 5, 10 and 20%

| **Sample ID** | **Prediction of using CXCR4 as a coreceptor** | | | **Progression** |
| --- | --- | --- | --- | --- |
|  | **FPR - 5%** | **FPR 10%** | **FPR 20%** |  |
| 1002 | R5 | R5 | R5 | Yes |
| 1013 | R5 | R5 | R5 | No |
| 1020 | R5 | R5 | R5 |  |
| 1022 | R5 | R5 | R5 | No |
| 1043 | R5 | R5 | R5 | No |
| 1044 | R5 | R5 | R5 | No |
| 1046 | X4 | X4 | X4 | Yes |
| 1047 | R5 | R5 | R5 | No |
| 1048 | R5 | R5 | R5 | No |
| 1049 | X4 | X4 | X4 | Yes |
| 1052 | R5 | R5 | R5 | No |
| 1053 | X4 | X4 | X4 |  |
| 1054 | R5 | R5 | R5 | Yes |
| 1055 | R5 | R5 | R5 | No |
| 1057 | R5 | R5 | R5 | No |
| 1060 | R5 | R5 | R5 | No |
| 1061 | R5 | R5 | R5 | Yes |
| 1064 | R5 | X4 | X4 | No |
| 1066 | R5 | R5 | R5 | No |
| 1067 | R5 | R5 | R5 | No |
| 1068 | R5 | R5 | X4 | No |
| 1070 | R5 | R5 | R5 | Yes |
| 1071 | R5 | X4 | X4 |  |
| 1072 | R5 | R5 | R5 | No |
| 1074 | R5 | R5 | X4 | No |
| 1075 | R5 | R5 | R5 | No |
| 1076 | R5 | R5 | X4 | No |
| 1077 | R5 | R5 | R5 | No |
| 1078 | R5 | R5 | R5 |  |
| 1079 | R5 | R5 | X4 | Yes |
| 1080 | R5 | R5 | R5 |  |
| 1081 | R5 | R5 | R5 | No |
| 1082 | X4 | X4 | X4 |  |
| 1084 | R5 | R5 | R5 | No |
| 1085 | R5 | R5 | R5 | No |
| 1089 | R5 | R5 | R5 | No |
| 1090 | R5 | R5 | R5 | No |
| 1092 | R5 | R5 | R5 | No |
| 1093 | R5 | R5 | R5 | Yes |
| 1094 | R5 | R5 | R5 |  |
| 1095 | X4 | X4 | X4 | No |
| 1097 | X4 | X4 | X4 |  |
| 1098 | R5 | R5 | R5 | No |
| 1100 | R5 | R5 | R5 | No |
| 1103 | X4 | X4 | X4 | No |
| 1104 | R5 | R5 | R5 | Yes |
| 1107 | R5 | R5 | R5 |  |
| 1108 | R5 | R5 | R5 | Yes |
| 1110 | R5 | R5 | R5 | No |
| 1111 | R5 | X4 | X4 | Yes |
| 1112 | R5 | R5 | R5 | No |
| 1114 | R5 | R5 | R5 | No |
| 1115 | R5 | R5 | X4 | No |
| 1117 | R5 | R5 | R5 | Yes |
| 1119 | R5 | R5 | R5 | No |
| 1120 | R5 | R5 | R5 | No |
| 1121 | R5 | R5 | X4 | Yes |
| 2015 | X4 | X4 | X4 |  |
| 2018 | R5 | R5 | R5 |  |
| 2019 | R5 | R5 | R5 | No |
| 2020 | R5 | R5 | R5 | Yes |
| 2022 | X4 | X4 | X4 | No |
| 2025 | R5 | R5 | R5 | Yes |
| 2027 | R5 | R5 | R5 |  |
| 2028 | R5 | R5 | R5 |  |
| 2029 | R5 | R5 | R5 | Yes |
| 2031 | X4 | X4 | X4 | Yes |
| 2032 | X4 | X4 | X4 | Yes |
| 2033 | X4 | X4 | X4 |  |
| 2036 | R5 | R5 | R5 | No |
| 2038 | R5 | R5 | R5 | No |
| 2040 | R5 | R5 | R5 |  |
| 2041 | R5 | R5 | R5 | No |
| 2042 | R5 | R5 | R5 | Yes |
| FPR - False Positive Rate | | | | |
